# An Enhanced Immune Response of *Mclk1* ^+/−^ Mutant Mice Is Associated with Partial Protection from Fibrosis, Cancer and the Development of Biomarkers of Aging

**DOI:** 10.1371/journal.pone.0049606

**Published:** 2012-11-14

**Authors:** Dantong Wang, Ying Wang, Catherine Argyriou, Audrey Carrière, Danielle Malo, Siegfried Hekimi

**Affiliations:** 1 Department of Biology, McGill University, Montreal, Canada; 2 Department of Medicine and Human Genetics, McGill University, Montreal, Canada; IGBMC/ICS, France

## Abstract

The immune response is essential for survival by destroying microorganisms and pre-cancerous cells. However, inflammation, one aspect of this response, can result in short- and long-term deleterious side-effects. *Mclk1* ^+/−^ mutant mice can be long-lived despite displaying a hair-trigger inflammatory response and chronically activated macrophages as a result of high mitochondrial ROS generation. Here we ask whether this phenotype is beneficial or simply tolerated. We used models of infection by *Salmonella* serovars and found that *Mclk1* ^+/−^ mutants mount a stronger immune response, control bacterial proliferation better, and are resistant to cell and tissue damage resulting from the response, including fibrosis and types of oxidative damage that are considered to be biomarkers of aging. Moreover, these same types of tissue damage were found to be low in untreated 23 months-old mutants. We also examined the initiation of tumour growth after transplantation of mouse LLC1 carcinoma cells into *Mclk1* ^+/−^ mutants, as well as during spontaneous tumorigenesis in *Mclk1* ^+/−^ *Trp53* ^+/−^ double mutants. Tumour latency was increased by the *Mclk1* ^+/−^ genotype in both models. Furthermore, we used the transplantation model to show that splenic CD8+ T lymphocytes from *Mclk1* ^+/−^ graft recipients show enhanced cytotoxicity against LLC1 cells in vitro. *Mclk1* ^+/−^ mutants thus display an association of an enhanced immune response with partial protection from age-dependent processes and from pathologies similar to those that are found with increased frequency during the aging process. This suggests that the immune phenotype of these mutants might contribute to their longevity. We discuss how these findings suggest a broader view of how the immune response might impact the aging process.

## Introduction

The immune system is essential for immediate survival in response to acute challenges from infection and cancerous cells. However, as is the case for other organs, the function of the immune system deteriorates during aging in a way that can be deleterious [1], [2], and markers of immunosenescence are inversely associated with long-term survival [3], [4]. The immune system has also been implicated in the aging process in a different way. It has been suggested that the activity of the immune system might in fact cause part of the phenotype observed in aged individuals [5]. In particular the process of inflammation, which is triggered by the innate immune system, has been implicated in triggering or exacerbating the development of chronic diseases of aging, including, type II diabetes [6], atherosclerosis [7], tumour growth [8], and all the metabolic consequences of obesity [9]. In addition, chronic inflammation leads to progressive fibrosis [10], a wound-healing response that involves the accumulation of extracellular matrix proteins and the cross-linking of extracellular collagen. Fibrosis is a hallmark of aging in a variety of organs, including the liver [11], and can lead to abnormal tissue structures and result in severe organ dysfunction. Moreover, it has been suggested that, in humans, episode of strong inflammation during infancy lead to long-term detrimental effects because of enhanced inflammatory processes in later life [12]. This hypothesis is based on the observation that, historically, declines in old-age mortality were preceded by declines in early-age mortality due to fewer episodes of severe infection and thus inflammation.

In contrast, our previous study of long-lived *Mclk1* ^+/−^ mouse mutants did not support the notion that inflammation triggers or worsens the aging process [13]. *Mclk1* (a.k.a. *Coq7*) encodes an enzyme involved in ubiquinone (UQ) biosynthesis [14], and loss of one copy of *Mclk1* (in *Mclk1* ^+/−^ mutants) results in i) altered UQ distribution in mitochondrial membranes [15], ii) altered function of the mitochondrial respiratory chain [16], iii) an increase in the generation of mitochondrial reactive oxygen species (mitROS) [16], iv) a decrease in the rate of development of oxidative biomarkers of aging [17], and v) an increased lifespan [17], [18]. In these mutants the increase in mitROS leads to increased expression of the immune regulator HIF-1α in macrophages [13]. HIF-1α has been found to be necessary for various aspects of immune function, including as a trigger of the differentiation of macrophages along the inflammatory pathway of activation [19]. Consistent with these observations we found that *Mclk1* ^+/−^ mutants had activated macrophages, and that the mutants reacted with a much stronger immediate elevation of circulating inflammatory cytokines upon treatment with very low levels of lipopolysaccharide (LPS) [13]. Thus, in *Mclk1* ^+/−^ mutants, in contrast to expectation, a high inflammatory reactivity was found to be associated with a slow development of biomarkers of aging and with an increased lifespan. An important question is whether the altered immune response of *Mclk1* ^+/−^ mutants could participate in bringing about the long-life phenotype.

To address this question, we have asked whether the enhanced immune response in *Mclk1* ^+/−^ mice is actually beneficial to the organism in terms of resistance to bacterial infection, cancer and the sort of markers of damage that are considered to be biomarkers of aging.

## Results

### *Mclk1* ^+/−^ Mutants Better Control Infections by *Salmonella* Typhimurium

**Figure 1:**
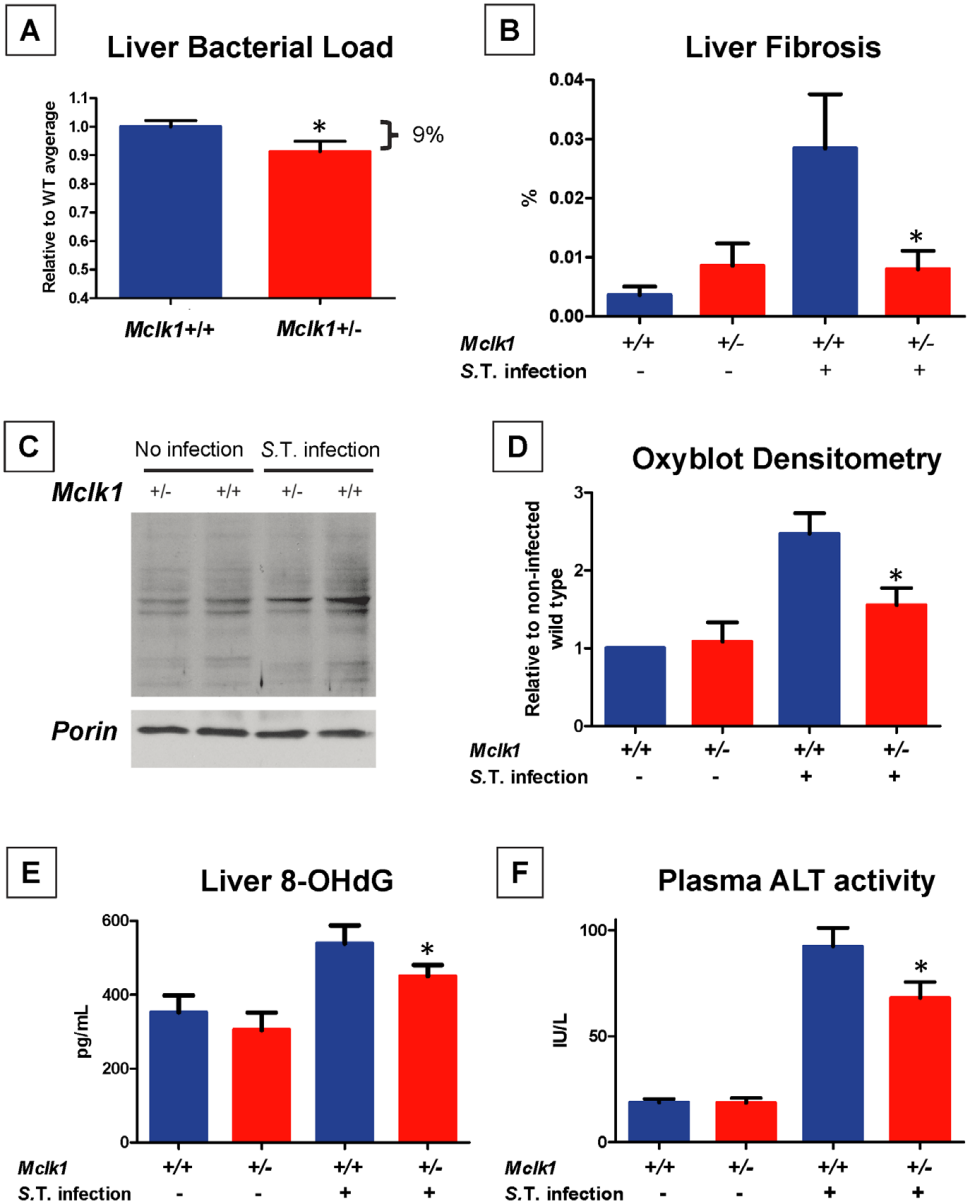
Favorable outcomes after infection of *Mclk1* ^+/−^ mutants with *Salmonella* Typhimurium. **A.** Liver bacterial loads were evaluated 40 days after infection in 3 months-old mice and were lower in *Mclk1* ^+/−^ mutants. Three independent experiments were carried out. In each experiment, the average CFU per gram of tissue of each mutant (*Mclk1* ^+/−^) and wild type animal (*Mclk1* ^+/+^) was normalized to the average of the wild type group in that experiment. In total 35 wild type animals and 23 mutants were normalized in this way and the results pooled. The mean ± S.E.M. of the pool is shown. * indicates p<0.05. **B.** Liver fibrosis was determined in infected and uninfected groups on day 40 after infection using Picrosirius Red staining. The fraction of the area of sections that stain for collagen is shown. The mean ± S.E.M. are shown. There were 9–14 animals in each group. **C.** and **D.** Oxidative damage to proteins was evaluated by the Oxyblot assay. All samples used were from littermate pairs. A representative result is shown in **C**. The intensity of staining in each lane was quantified and normalized by that of the wild type in the same blot. The mean ± S.E.M. of each group is shown on the graph and paired t-tests were used for comparisons. **E.** Oxidative damage to DNA was determined by measuring 8-OHdG levels using purified total liver DNA. Littermate pairs were used (*Mclk1* ^+/−^ vs. *Mclk1* ^+/+^): 5 pairs without infection and 6 pairs sacrificed 40 days after infection. The mean ± S.E.M. of each group is shown in the graph; paired t-test were used for comparisons. **F.** Plasma ALT activity was measured 40 days after infection. There were 8–14 animals in each group and the mean ± S.E.M. of each group are shown. For **B−F**, the differences between the mutant and wild type groups are significant with, but not without, infection at p<0.05. All experiments in this figure were carried out with males.

We tested the performance of *Mclk1* ^+/−^ mutants in a model of infection by *S*. Typhimurium. We used *Mclk1* ^+/−^ mutants and sibling wild type controls on the 129S6/SvEvTac (129S6) genetic background. Mice with this background are wild type at the *Slc11a11* locus and can survive *S*. Typhimurium infection, but cannot clear the bacteria completely and thus develop a chronic infection [20]. We injected mutants and wild type controls intravenously at a dose of 1140 CFU per mouse and scored liver bacterial load (CFU per gram tissue) after 40 days. The mutants show a small but statistically significant decrease of ∼9%, indicative of a somewhat more efficient immune control of *S.* Typhimurium infection (Figure 1A).

### *Mclk1* ^+/−^ Mutants Sustain Less Tissue Damage in Response to *S.* Typhimurium Infection

Bacterial infection and the immune reaction against it leads to tissue damage, including oxidative damage. In liver samples of the same animals as in Figure 1A we also tested fibrosis (Figure 1B), oxidative damage to proteins, using oxyblot (Figure 1C and 1D), and oxidative damage to DNA, by measuring the levels of 8-OHdG (Figure 1E). In the plasma sample from these animals we measured alanine amino-transferase (ALT), a measure of damage to liver cells (Figure 1F). There was a large increase in all four measures in infected animals in comparison to uninfected controls. However, the increase was significantly less marked in mutant animals for all four measures. In fact, in the case of fibrosis there was no increase at all. The levels of fibrosis were very low but the difference between the genotypes after infection is significant nonetheless, and is also consistent with other findings on sensitivity to fibrosis in the *Mclk1* ^+/−^ mutant mice (see below). Note that in this experiment we cannot ascertain whether the lower level of damage is due to the reduced bacterial load or a change in the level of damage produced by the immune response itself.

### *Mclk1* ^+/−^ Mutants Display an Altered Immune Reaction to *S.* Enteritidis Infection

**Figure 2:**
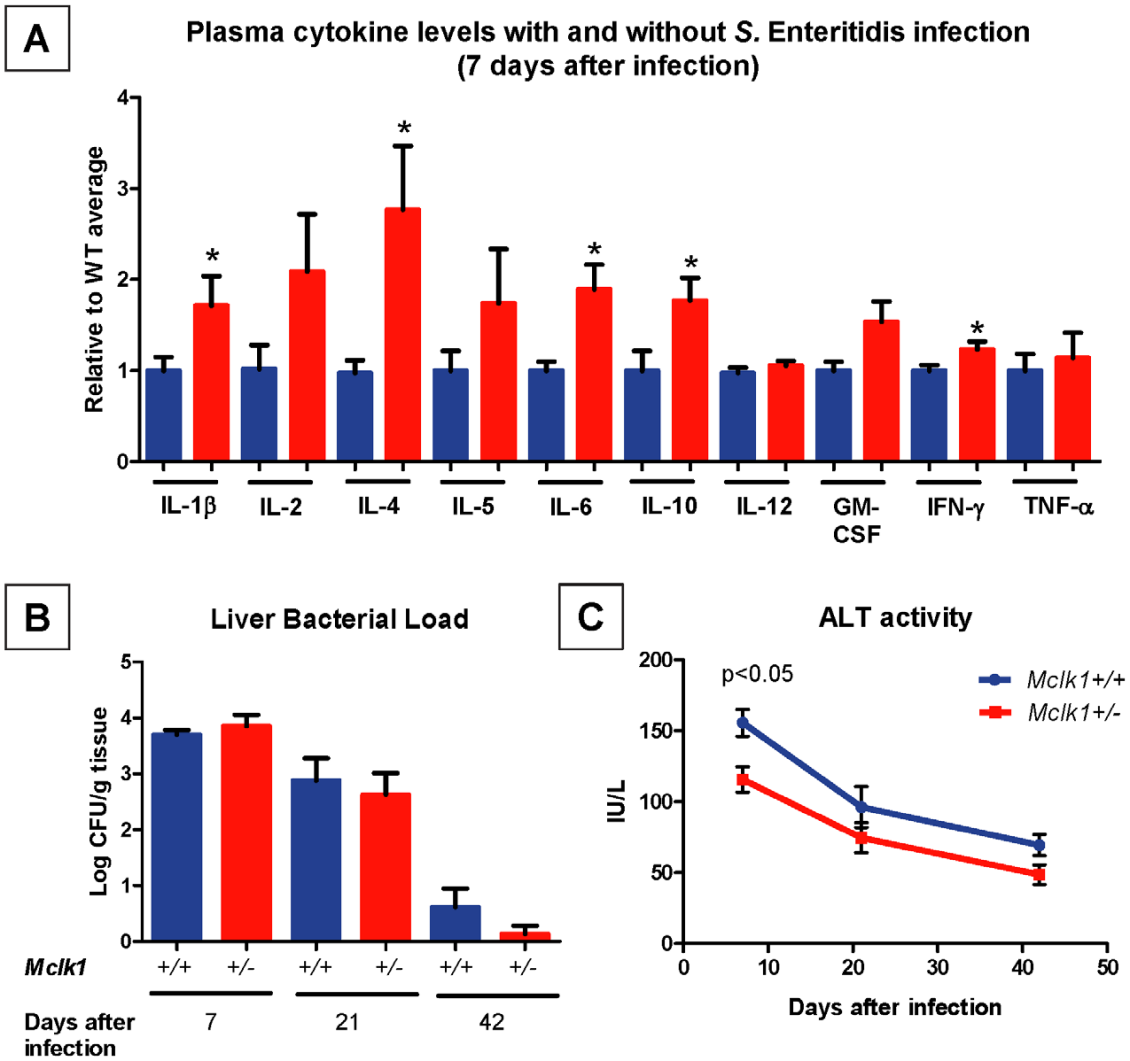
Enhanced immune response and lower tissue damage after *Salmonella* Enteritidis infection of *Mclk1^+/−^* mutants on the C57BL/6J background. A. Three months-old littermates of both genotypes were infected intravenously with 1800 CFUs per mouse, their plasma collected after 7 days and evaluated for cytokine levels. For each cytokine the values were normalized to the mean value measured for the *Mclk1* ^+/+^ control group. The sample size was 32–38 for each genotype. The asterisks represent p<0.05. **B.** Three months-old mice on the C57BL/6L background were allocated into 3 groups of 9–12 mice for each genotype. Each mouse was infected intravenously with 10^3^ CFUs *S.* Enteritidis. Bacterial loads in liver were measured at days 7, 21 and 42 after infection. C. Plasma ALT activity was measured on day 7, 21 and day 42 after infection. 5–13 mice in each genotypic group at each time point were used, and the mean ± S.E.M. is shown. All experiments in this figure were carried out with even numbers of both males and females.

Our original observations that the immune system is affected by high mitochondrial oxidative stress in *Mclk1* ^+/−^ mutants was carried out on the C57BL/6J background [13]. For example, it is on that background that we observed that the mutants responded to a low level of LPS treatment with a much greater elevation of circulating plasma cytokines. However, *S.* Typhimurium infections are lethal on the C57BL/6J background, which bears a loss-of-function mutation at the *Slc11a11* locus [21]. In contrast, experimental intravenous infections with a different serovar, *Salmonella* Enteritidis, can be completely cleared from the liver 40 days after infection on the C57BL/6J background [22]. To link findings with experimental infections to our previous work on this background, we first tested whether the immune reaction to *S.* Enteritidis was different in the mutants, as it was to LPS. Using an assay (Luminex) by which the level of 10 plasma cytokines can be simultaneously assessed, we determine cytokine levels 7 days after infection with *S.* Enteritidis (Figure 2A). We found that both pro-inflammatory Th1 cytokines (IL-1β, IL-6, and IFN-γ) and anti-inflammatory Th2 cytokines (IL-4 and IL-10) were elevated compared to controls. In addition, IL-2, IL-5 and GM-CSF showed a clear trend toward a similar elevation. Overall, these findings indicate that several aspects of the immune reaction to *S.* Enteritidis infection in *Mclk1* ^+/−^ mutants are more pronounced than in the wild type.

To determine if the different reactivity to the infection as witnessed by higher cytokine levels was altering the clearance of the bacteria from the liver we determine the bacterial load at 7, 21 and 42 days after the infection (Figure 2B). No significant difference between mutant and wild type bacterial load was observed at any time following infection. However, in spite of similar bacterial load, damage to the liver, as measured via plasma ALT levels, was reduced on day 7 in *Mclk1* ^+/−^ mutants and showed the same trend toward reduction on the other days (Figure 2C).

### *Mclk1* ^+/−^ Mutants Sustain a Slower Increase of Tissue Damage Over Time and in Response to *S.* Enteritidis Infection

**Figure 3:**
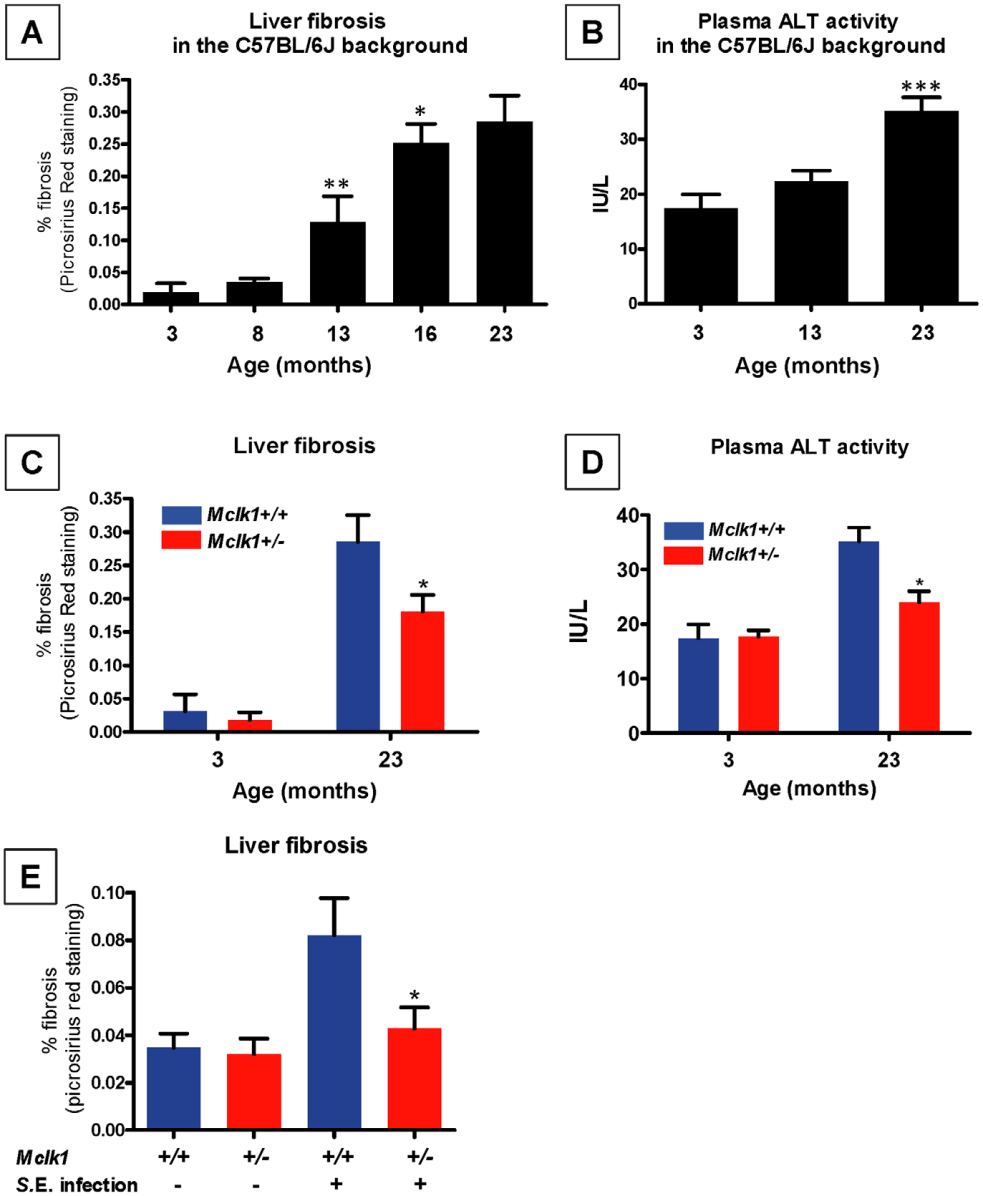
Favorable effects of the *Mclk1* ^+/−^ genotype on fibrosis and plasma ALT levels during aging and after repeated *S.* Enteritidis infection. **A.** The Level of liver fibrosis in wild type C57BL/6J mice of different age was determined. Sample sizes were from 5 to 16 and the means ± S.E.M. of each age group is shown. The asterisks indicate statistical significance compared to the immediately younger age group by a t-test. * stands for P<0.05, ** for P<0.01, and *** for P<0.001. **B.** Plasma ALT activity of wild type C57BL/6J mice was measured and the mean ± S.E.M. of each age group is shown. Sample sizes were from 5 to 14. **C.** The level of liver fibrosis in young and old *Mclk1* ^+/−^ mutants on the C57BL/6J background and their *Mclk1* ^+/+^ siblings was determined. Sample size was 5 for the 3 months-old cohort and from 17 to 20 for the 23 months-old cohort. **D.** ALT activity in *Mclk1* ^+/−^ mutants and *Mclk1* ^+/+^ siblings on the C57BL/6J background was measured at 3 months and 23 months of age. There were 5 mice for each genotype in the 3 months-old cohort, and from 15 to 16 mice in the 23 months-old cohort. **E.** The level of liver fibrosis was determined after 3 rounds of *S.* Enteritidis infection. There were 11–16 mice in each group; the mean ± S.E.M. of each group is shown. The asterisks indicate statistical significance compared to the wild type group by a t-test. * stands for P<0.05. All experiments in this figure were carried out with even numbers of both males and females.

In a previous study, we observed that the gradual development of mitochondrial and oxidative biomarkers of aging was slowed in *Mclk1* ^+/−^ mutants compared to sibling *Mclk1* ^+/+^ controls [17]. In order to continue our studies in the C57BL/6J background we first investigated whether some of the markers of damage that we had observed to be lower following *S.* Typhimurium infection behaved as biomarkers of aging in C57BL/6J mice. For this we first determined that liver fibrosis, a measure of cumulative tissue damage, and plasma alanine aminotransferase (ALT) activity, a measure on ongoing liver damage [23], increased with age in this background (Figure 3A and 3B). We then measured fibrosis and ALT in young (3 months-old) and old (23 months-old) mutants and controls on the C57BL/6J background (Figure 3C and 3D). For both markers there was no difference in young animals but a marked reduction in the mutants compared to controls was observed in old animals.

One hypothesis to explain the low damage in old *Mclk1* ^+/−^ mutants is that it results from a lifetime of less damaging immune responses to infections and other inflammatory processes (as suggested by the experiments with *S.* Typhimurium). To further test this idea we took advantage of the fact that *S.* Enteritidis is fully cleared from the liver in less than two months in the C57BL/6J background [22]. We therefore carried out a repeated-infection study in which mice were infected 3 times at 2 months interval starting at 3 months of age, and plasma ALT as well as liver fibrosis were measured one month after the last infection. As shown in Figure 3E, liver fibrosis in the wild type was dramatically elevated compared to age-matched uninfected controls. The level of damage observed was in the range of that observed in uninfected, but older, 13 months-old mice (Figure 3A), thus confirming that liver fibrosis is a cumulative damage marker that is accelerated by infection. In contrast to the wild type situation, *Mclk1* ^+/−^ mutants were unaffected by the 3 rounds of infection (Figure 3E), reinforcing our interpretation that the lower level of fibrosis in uninfected old *Mclk1* ^+/−^ mutants (Figure 3C) could be the result of a lifetime of less damaging immune responses, which in turn could be part of what allows for the mutants’ longevity. Future studies to understand the low fibrotic response in *Mclk1* ^+/−^ mutants should involve the study of signaling through TGF-β, a molecule that is frequently associated with the regulation of fibrosis.

In contrast to fibrosis, ALT levels were completely unaffected after the three rounds of infection (not shown). This is consistent with what we had observed after a single round of infection by *S.* Enteritidis (Figure 2C), when ALT levels, which were lower in *Mclk1* ^+/−^ mutants immediately following the infection were back to normal 40 days later. This is in contrast to what we observed with *S.* Typhimurium infection, which is not cleared but remains chronic, and where ALT levels are still strongly elevated after 40 days (Figure 1F). This confirms that ALT is not a measure of accumulating damage but rather of ongoing damage. These findings also suggest that the high ALT levels observed in old wild type animals (Figure 3D) is a measure of ongoing pathological processes.

### Mitochondrial Oxidative Stress Per Se does not Stimulate the Immune Response Nor Reduce Fibrotic Damage

**Figure 4:**
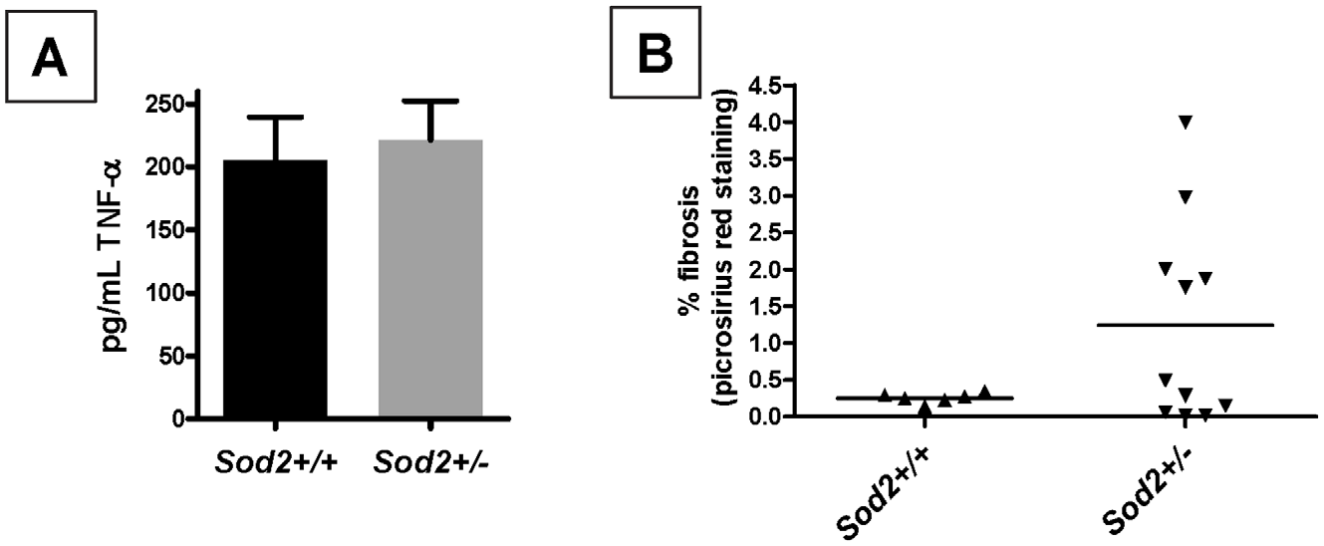
The reaction to LPS and fibrosis in *Sod2* ^+/−^ mutants are different from those in *Mclk1* ^+/−^ mutants. **A.** TNF-α levels in the plasma of 3 months-old *Sod2* ^+/−^ mutants and controls two hours after IP injection of 0.01 mg/kg LPS, measured by ELISA. A t-test did not indicate any difference in TNF-α levels between *Sod2^+/−^* mutants and controls (n = 15−17; error bars represent S.E.M. ). **B.** The level of liver fibrosis in *Sod2* ^+/−^ mutant males and controls at 16 months of age was determined. There were 6 and 11 mice in the *Sod2* ^+/−^ and *Sod2* ^+/+^ groups, respectively. All experiments in this figure were carried out with males only.

Mutants heterozygous for a knockout of the mitochondrial superoxide dismutase gene *Sod2,* which are fully viable, display elevated mitochondrial oxidative stress like *Mclk1* ^+/−^ mutants [17], [24], [25]. We investigated whether the effect of the *Mclk1* ^+/−^ mutation on immune responsiveness and damage was also induced in *Sod2* ^+/−^ mutants. *Mclk1* ^+/−^ mutants react to very low levels of LPS (0.01 mg/kg) with a dramatic elevation of serum cytokine levels, in particular TNF-α, which was observed by the multiple-cytokine Luminex test as well as by a TNF-α-specific ELISA [13]. We tested 3 months-old *Sod2* ^+/−^ mutants for reactivity to very low level of LPS and found no elevation of plasma TNF-α compared to the wild type controls (Figure 4A). We also tested the level of liver fibrosis in aged mutants (16 months-old) (Figure 4B). There was no decrease in fibrosis in the mutants, but a strong trend suggesting a dramatic increase in fibrosis. These findings are consistent with the hypothesis that the reduction in age- and infection-dependent damage, in particular fibrosis, observed in *Mclk1* ^+/−^ mutants result from their enhanced immune response, rather than from other physiological changes that can be induced by elevated mitochondrial oxidative stress.

### Indirect Evidence for Delayed Tumor Initiation and/or Growth in *Mclk1* ^+/−^ Mutants

**Figure 5:**
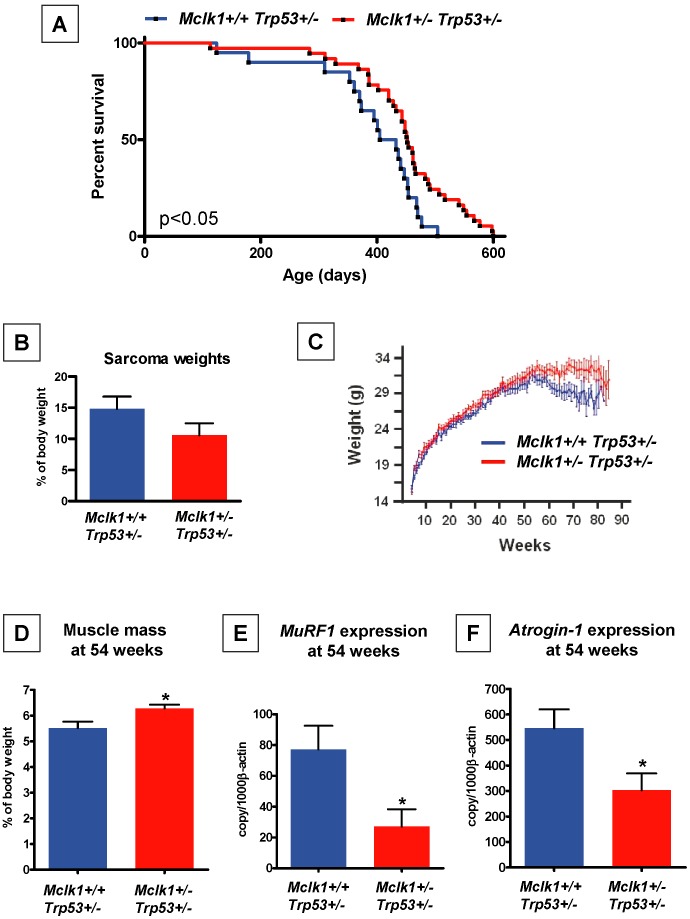
Indirect evidence for altered tumorigenesis in the *Mclk1* ^+/−^ mutants. **A.** Kaplan-Meier survival curves of *Mclk1* ^+/+^ *Trp53* ^+/−^ (n = 20) and *Mclk1* ^+/−^ *Trp53* ^+/−^ (n = 37) mice. The significance of the difference was P<0.05 by the log-rank (Mandel-Cox) test using GraphPad Prism Version 5.00 for Windows (GraphPad Software, San Diego). **B.** Weights of spontaneous sarcoma scored at necropsy of the animals in A., and expressed as percentage of body weight; *Mclk1* ^+/+^ *Trp53* ^+/−^ (n = 9) and *Mclk1* ^+/−^ *Trp53* ^+/−^ (n = 19). **C.** Body weights over time of the animals in A. **D.-F.** A cohort distinct from the animals in A. was analyzed for signs of cachexia; *Mclk1* ^+/+^ *Trp53* ^+/−^ (n = 12) and *Mclk1* ^+/−^ *Trp53* ^+/−^ (n = 8). All mice were humanely euthanized at 54 weeks of age. Hind limb muscle weight were measured and expressed as relative to body weight (**D**). The mRNA expression levels of two muscle specific E3 ubiquitin ligases, Atrogin-1 and MuRF1, were measured by RT-PCR, shown in (**E**) and (**F**), respectively. Significance for the aging curves was established by a log-rank test. The significance levels for the data in B. and D.-F. were tested by t-tests, with the asterisks standing for p<0.05. All experiments in this figure were carried out with females only.

Evaluating any effect of the *Mclk1* ^+/−^ mutant genotype on cancer is interesting for two reasons: *i*) cancer is clearly an age-dependent disease and *ii*) the cancer rate is believed to be kept low by mechanisms of immune surveillance. To test the possibility that the phenotype of *Mclk1* ^+/−^ mutants affects cancer development we constructed a *Mclk1* ^+/−^ *Trp53* ^+/−^ double mutant strain on the C57BL/6J background. *Trp53* ^+/−^ mutants spontaneously develop tumors, in particular sarcomas, after approximately one year [26]. We produced groups of *Trp53* ^+/−^ (n = 20) and *Mclk1* ^+/−^ *Trp53* ^+/−^ (n = 37) females by crossing *Trp53* ^−/−^ males to *Trp53* ^+/−^ *Mclk1* ^+/−^ females. We examined the survival of these animals and measured the size of visible sarcomas at the time of death. The average and maximum lifespan of the double mutants was increased in comparison to *Trp53* ^+/−^ animals (Figure 5A), suggestive of a slower initiation or growth of tumors. However, the overall incidence of sarcoma development was comparable between the two genotypes (not shown) and the weight of the sarcomas that could be identified during necropsy showed only a trend toward smaller tumors in the double mutants (Figure 5B). However, these results might still point to slower tumor initiation or growth because tumors were scored at necropsy and double mutants survived longer, implying that the animals had more time to develop tumors sufficiently large to score.

*Trp53* ^+/−^ mutants abruptly started losing weight after 54 weeks, presumably because of tumor-induced cachexia, but this effect was much less severe in the double mutants (Figure 5C). To examine this question further we produced two additional groups of *Trp53* ^+/−^ (n = 12) and *Mclk1* ^+/−^ *Trp53* ^+/−^ (n = 8) double mutants. At 54 weeks of aged, immediately before the observed beginning of weight loss in the initial experiment, we examined parameters associated with cachexia, such as muscle weight and the expression of cachexia-associated genes in muscles, such as those coding for the muscle-specific ubiquitin ligases MuRF1 (*Trim63*) [27] and Atrogin-1 (*Fbxo32*) [28] (Figure 5E–F). Muscle weight was higher in the double mutants, and *MuRF1* and *Atrogin-1* gene expression was lower. Note that the slower development of cachexia is consistent with the notion of a slower initiation and growth of tumors.

### Direct Evidence for Delayed Tumor Initiation in *Mclk1* ^+/−^ Mutants

**Figure 6:**
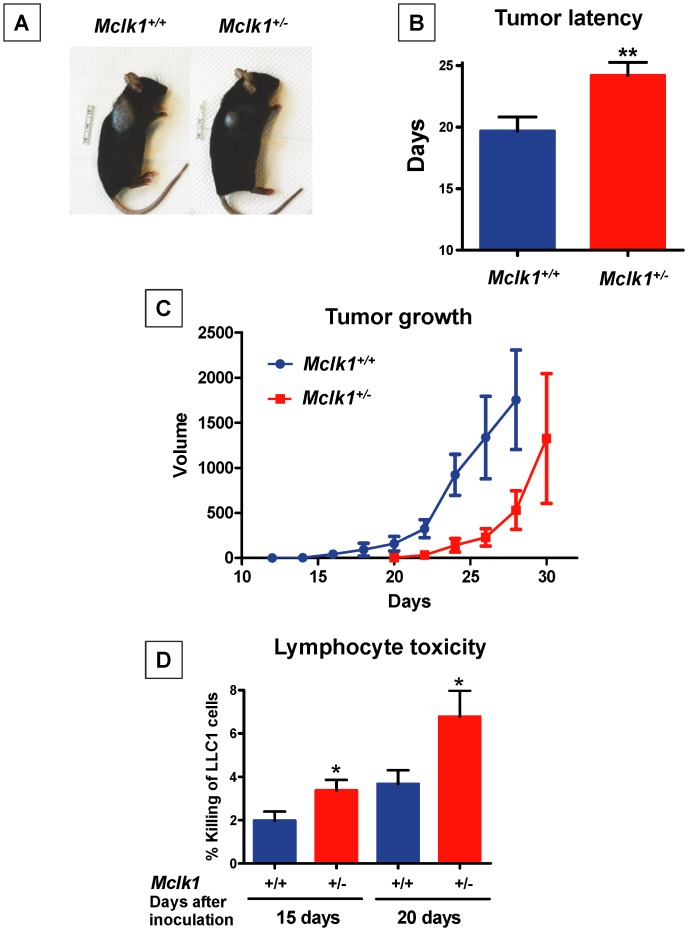
Direct evidence for altered tumorigenesis in the *Mclk1* ^+/−^ mutants. **A.** LLC1 mouse lung cancer cells were injected subcutaneously in the right supra-scapular area of 10-week-old *Mclk1* ^+/−^ mutants (n = 19) and sibling controls (n = 18) in the C57BL/6J background at a dose of 10^4^ cells/mouse. The time of first appearance of visible tumors was recorded and their growth subsequently followed. The panel illustrates tumor sizes on day 28 after inoculation. **B.** Average day of tumor appearance after grafting in both genotypes. The asterisks indicate significance at P<0.01 by a t-test. **C.** Tumor growth curves reveal again the difference in latency but also show similar growth rates once tumors have appeared. Data after day 28 are not available for tumors on wild type animals because these animals had to be humanely euthanized due to tumor size. **D.** Two cohorts of 8–12 mice for each genotype were used to measure splenic lymphocyte cytotoxicity against LLC1 cells in vitro after 15 and 20 days following inoculation. The mean ± S.E.M. of each group is shown. The asterisks indicate significance at p<0.05 by a t-test. All experiments in this figure were carried out with even numbers of both males and females.

To obtain more direct evidence for an effect of *Mclk1* ^+/−^ on tumor initiation and growth we used a model [29] in which 10^4^ Lewis lung carcinoma cells (LLC1 cells, a.k.a. 3LL cells) were grafted subcutaneously into 3 months-old mutants and controls (Figure 6A). With this model we found that tumor initiation was significantly delayed in *Mclk1* ^+/−^ mutants (Figure 6B). A trend toward lower tumor incidence was also observed but was not significant (not shown). However, once a visible tumor had appeared the tumor growth rate was not different in the two genotypes (Figure 6C).

### Enhanced Immune Reaction Against Tumor Cells in *Mclk1* ^+/−^ Mutants

We sought to determine whether immune function played a role in the tumor latency phenotype of *Mclk1* ^+/−^ mutants observed in the grafting model. Cytotoxic CD8+ T cells are believed to participate in the immune reaction against cancerous cells [30], [31]. Thus, we extracted splenic lymphocytes from graft hosts, isolated CD8+ T cells by magnetic bead separation and tested their cytotoxicity against LLC1 cells in vitro [29], [32]. After both 15 and 20 days following grafting the cytotoxicity of the lymphocytes from *Mclk1* ^+/−^ was greater than that of the controls (Figure 6D). At day 15 and day 20 there were no or only very small tumors on these animals (similarly to what we observed in the cohort shown in Figure 6C). This suggests that the difference in immune system reactivity against the grafted cells, leading to more, or more active, cytotoxic effector cells could be responsible for the delay in tumor initiation.

## Discussion

We have shown previously that *Mclk1* ^+/−^ mutants sustain high mitochondrial oxidative stress as young animals [16], [17]. This was a surprise because *Mclk1* ^+/−^ mutants age slowly, as assessed by biomarkers of aging, and can be long-lived, while mitROS have generally been considered to be deleterious and possibly a cause of aging. In light of the numerous findings that ROS are signaling molecules and that animals like naked mole-rats can be long-lived in spite of elevated oxidative stress [33], [34], we have interpreted our findings to mean that mitROS do not cause aging but might instead be associated with aging because they act as signaling molecules that turn on protective mechanisms in reaction to age-dependent molecular damage [35], [36]. In our subsequent search for mitROS-dependent protective mechanisms we uncovered that the immune system of *Mclk1* ^+/−^ mutants was activated and highly reactive, possibly via the stabilization of HIF-1α by mitROS in liver and in macrophages and possibly in other tissues [13].

The high mitochondrial oxidative stress in *Mclk1* ^+/−^ mutants cannot in itself be responsible for their favorable phenotype, as lowering oxidative stress in the mitochondria can produce beneficial phenotypes [37], and as *Sod2* ^+/−^ mutants, which have a mitochondrial phenotype very similar to that of *Mclk1* ^+/−^ mutants [17], do not show the same slow development of biomarkers of aging [17]. Here we show in addition that *Sod2* ^+/−^ mutants, in contrast to *Mclk1* ^+/−^ mutants, do not show an exaggerated elevation of plasma TNF-α in response to LPS, nor low fibrosis (Figure 4). The difference between the effects that high mitochondrial oxidative stress has in *Mclk1* ^+/−^ and *Sod2* ^+/−^ mutants might be linked to the role of MCLK1 in UQ biology. Indeed, we recently found that *Mclk1* ^+/−^ mutants have low UQ levels in the inner mitochondrial membrane, which is likely responsible for the mitochondrial phenotype, but also show elevated UQ levels in the outer mitochondrial membrane, which might alter how mitROS signal to the rest of the cell [15].

Here we have asked whether the altered immune response in *Mclk1* ^+/−^ mutants is in fact protective and thus could participate in promoting longevity. In our previous study [13] we mostly explored inflammation. For example, we tested the effect of LPS on circulating cytokines two hours after the treatment. We also asked whether peritoneal macrophages were differentiated preferentially along the classical inflammatory path of differentiation (as contrasted to the alternative path of differentiation that favors fibrosis). To establish whether the fast inflammatory response observed in *Mclk1* ^+/−^ mutants was their principal immune phenotype or whether their immune response was more profoundly altered, we infected *Mclk1* ^+/−^ mutants with *S.* Enteritidis and let the infection proceed for 7 days, at which time we examined circulating cytokines (Figure 2A). With this more complex stimulus (live bacteria instead of LPS) and letting the response progress for 7 days we now observed that both pro-inflammatory cytokines (IL-1β, IL-6, and IFN-γ) and anti-inflammatory cytokines (IL-4 and IL-10) were significantly elevated compared to controls. In addition, IL-2, IL-5 and GM-CSF showed a strong trend toward elevation. Thus, the enhanced immune reaction in *Mclk1* ^+/−^ mutants is not confined to inflammation but engages various aspects of the immune response.

Changes in cytokine levels cannot in themselves predict the outcome of an immune challenge. We therefore challenged the mutants using two distinct models of infection by *Salmonella* serovars. Infection with *S.* Typhimurium of mice on a 129S6 genetic background is a model of chronic infection with persistence of the bacteria. Infection with *S.* Enteritidis of animals on a C57BL/6J genetic background is a model of more short-term infection where the bacteria are rapidly cleared from tissues. There was a modest reduction in liver bacterial load with *S.* Typhimurium in *Mclk1* ^+/−^ mutants (Figure 1A) but no effect of the mutation on the rate of clearance with *S.* Enteritidis (Figure 2B). However, the main finding with these models was a remarkable reduction of markers of liver damage following infection of *Mclk1* ^+/−^ mutants, including reduction in fibrosis, oxidative damage to proteins, oxidative damage to DNA, and general stress to hepatocytes (measured by plasma ALT activity). Although uncontrolled bacterial proliferation is of course capable of inducing extensive damage of various types, it is likely that much of the damage we have been scoring are secondary effects of the immune response induced by the bacteria. The fact that *Mclk1* ^+/−^ mutants control infection better in one of the models and display less damage from infection in both models indicates that the more extreme immune response of the mutants is in fact beneficial to the outcome of infection.

Fibrosis, carbonyls, 8-OHdG, and ALT are biomarkers of aging, that is, they increase with chronological age without being specifically generated by a particular age-dependent disease. We have previously shown that *Mclk1* ^+/−^ mutants develop a number of biomarkers of aging more slowly, including carbonyls and 8-OHdG, as well as others, such as isoprostanes [17]. To help interpret the findings of low biomarkers of damage following infection, we first tested whether fibrosis and ALT were in fact biomarkers of aging in our hands and whether *Mclk1* ^+/−^ mutants were slower at developing these biomarkers. Figure 3 shows that both are true: plasma ALT and fibrosis increase gradually with age in wild type animals, and are significantly lower in *Mclk1* ^+/−^ mutants than in controls at 23 months of age.

Fibrosis is a scarring process that can results from the tissue damage brought about by the immune response [10]. Mild fibrosis in young animals is mostly resolved but extensive fibrosis observed in aged animals might be partly due to the accumulation of fibrotic tissue over time. Extensive fibrosis impacts on the proper function of the affected tissues and is reported to contribute to the aged phenotype. We wondered whether repeated non-lethal infection over time could be the cause of the gradual increase in liver fibrosis that we observed. To test this we subjected mice of both genotypes to three rounds of infection with *S.* Enteritidis. After each round the mice of both genotypes had completely cleared the infection, yet the wild type level of fibrosis more than doubled compared to mock-infected controls (Figure 3E). Most interestingly there was no increase in fibrosis in *Mclk1* ^+/−^ mutants in spite of the fact that the mutants do not control bacterial proliferation better than the controls (Figure 2B). We conclude that the lower level of fibrosis in old *Mclk1* ^+/−^ mutants might be the result of a lifetime of sustaining less fibrosis resulting from infections.

It is unclear whether the same reasoning we used for fibrosis can be used for the other biomarkers. Indeed, carbonyls and 8-OHdG are oxidized constituents of molecules (proteins and DNA) that can be repaired or turned over, and ALT marks damage to liver cells that can be replaced by regeneration. Thus, for these biomarkers the elevated levels observed in older animals are indicative of a higher steady state equilibrium between damage and repair. Our observation that these biomarkers are lower in *Mclk1* ^+/−^ mutants following infection as well as lower in 23 months-old animals suggest an immune origin of the generation of these markers. Indeed, an increase in the rate of infection is a particularly deleterious phenotype of aging. Possibly, an enhanced immune response allows the old mutants to suffer fewer or less severe ongoing chronic infections.

We suggest that the higher levels of fibrosis and of the other biomarkers of damage are due to the immune reaction against infection. Some might wonder where these infections come from in animal facilities that are described as “pathogen-free”. However, pathogen-free means that the facility is free of known transmissible pathogens that induce obvious and severe pathologies, not that the animals are free of all potentially infectious microorganisms or viruses.

*Mclk1* ^+/−^ mutants appear to be able to delay tumor development in two very distinct experimental settings (spontaneous tumor development in the *Trp53* ^+/−^ background, and after grafting of isogenic tumor cells). In the grafting model we have furthermore found that the transplanted tumor cells were capable of inducing a more powerful immune response, as measured by the cytotoxicity of splenic T cells against the tumor cells in vitro (Figure 6D). These findings suggest that the altered immune response of *Mclk1* ^+/−^ mutants also includes enhanced immune surveillance, which in turn allows for the tumor latency phenotype. Future studies targeting partial *Mclk1* inactivation to the immune system should help to further characterize the immune mechanisms behind this phenomenon.

Our findings with experimental infection and tumorigenesis, as well as on the development of biomarkers of aging, suggest that a globally enhanced immune response can be beneficial. Observations with human patients suggest that a susceptibility to infection, cancer, and gradual fibrosis are among the age-dependent processes that limit lifespan. Thus the effect on these processes of the enhanced immune response of *Mclk1* ^+/−^ mutants might in fact participate in allowing the long lifespan that has been observed with these mutants [17], [18]. Interestingly, this hypothesis suggest that the inevitable limitations on the salubriousness of animal facilities might participate in the comparative longevity of the mutants. In fact a similar mechanism might be at play in studies in which mutant mice have been found to be long-lived but only in comparison to relatively short-lived control mice and a subsequent study with higher quality animal husbandry found a much smaller difference between mutants and controls [38], [39].

Our hypothesis that an enhanced immune response might be beneficial for aging and lifespan raises a classical question that applies to any long-lived mutant: what is the trade-off? If the immune system can be stronger in a way that is not deleterious, why do not all mice have a stronger immune system? The answer is straightforward as the high energetic and fitness costs of the immune response are well known [40], [41]. However, in the environment of caged animals, where acquiring food and drink and mates is highly facilitated, an enhanced immune response as in *Mclk1* ^+/−^ mutants might only rarely be deleterious for immediate survival, but could slow down age-dependent pathologies and limit the accumulation of damage such as fibrosis and thus prolong lifespan, as our observations suggest.

Our findings and our hypothesis are not inconsistent with the other proposed ways in which immune system function might be involved in the aging process. For example, the fact that a high inflammatory status might be inversely correlated with long-term survival and the incidence of age-dependent diseases does not mean that a more powerful immune response that is not particularly tilted toward inflammation must be deleterious. In fact, the observations described in the present study, including the complex cytokine response to experimental bacterial infection, and the lesser immediate damage resulting from such infection in the mutants suggest that there is a moderation of the consequences of inflammation in these animals. Similarly, we have no information at the present time as to whether immunosenescence is impacted by the mechanisms at work in *Mclk1* ^+/−^ mutants. These mechanisms might slow down, accelerate, or have no effect on immunosenescence. If these mechanisms were to accelerate immunosenescence the moderate lifespan increase that can be observed in *Mclk1* ^+/−^ mutants might simply represent a new equilibrium between beneficial and deleterious effects on aging by the immune system.

## Materials and Methods

### Animals

*Sod2* ^+/−^ mice on a C57BL/6J background were purchased from Jackson Laboratories (stock #002973). *Trp53* ^+/−^ mice (B6.129-*Trp53^tm1Brd^* N12) on a C57BL/6J background were obtained from Taconic Farms (Germantown, NY). All mice were housed in the Animal Care Facility of McGill University. Unless otherwise specified, age-matched controls were used for all experiments. All animal experiments were performed under conditions specified by the Canadian Council on Animal Care and the animal use protocol was approved by the McGill University Facility Animal Care Committee.

### Bacteria and Infection

Two *Salmonella* strains were used for infection. *S.* Typhimurium (strain Keller) was used to infect 3 months-old *Mclk1^+/−^* male mice on the *Salmonella*-resistant 129S6 background, whereas 3 months-old C57BL/6J mice were infected with *S.* Enteritidis (strain 3b). Infections were performed using standard protocols as described [22]. In brief, *Salmonella* were grown to log phase in trypticase soy broth (Sigma) at 37°C, diluted in phosphate-buffered saline (PBS) and injected intravenously into the lateral tail vein. Mice in the infected groups received a single dose of 1140 CFU in 200 µl of PBS, except one group of C57BL/6J mice which was given 1800 CFU of *S.* Enteritidis for cytokine measurements at day 7 after infection. Infectious doses were verified by plating of serial dilutions on trypticase soy agar. Mock-infected mice were injected with PBS alone. In addition to a single dose infection, the present study also examined liver fibrosis after 3 rounds of Enteritidis infections at 2 months intervals. In the latter case, mice were euthanized one month after the last infection. *For measurement of bacterial load,* liver tissue (1.0 g) was homogenized in 2 mL of sterile PBS and serial dilutions were plated onto trypticase soy plates. After 24 h of incubation at 37°C, colonies were counted and expressed as log CFU per gram of tissue.

### Assessment of Liver Fibrosis

Liver samples, collected at different times after infection, were formalin-fixed, embedded in paraffin, sectioned (5 µm), and stained with Picrosirius Red (PSR) using standard methods. Briefly, sections were incubated for 1 hour in 0.1% Sirius red (Sigma) in saturated aqueous picric acid (Sigma). After rinsing in 0.5% acetic acid, sections were dehydrated, cleared in xylene and mounted for light microscopy. The PSR-stained sections were examined under polarized light with a Zeiss Axiovert 200 Inverted microscope and digitally captured images were analyzed with Image-Pro Plus software (Media Cybernetics) to quantify collagen fiber content. At least 3 sections were prepared per mouse liver and for each section 15 randomly selected microscopic fields were examined. The degree of fibrosis was calculated as the fraction of positively stained pixels relative to the total pixels.

### Markers of Oxidative Damage

Protein oxidation was determined by quantifying the carbonyl protein content of liver homogenates using the OxyBlot kit (Millipore, Billerica, MA) according to the manufacturer's recommendations. The 8-OHdG ELISA kit from Cayman Chemical was used to quantify oxidative DNA damage.

### Measurements of Plasma Cytokine Levels and ALT Activity

IL-1β, GM-CSF, INF-γ, IL-2, -4, -5, -6, -10, and -12, and TNF-α were measured from heparinised plasma using the Luminex multiple cytokine assay according to the manufacturer’s instruction (Invitrogen). ALT activity was measured by a kinetic assay using the ALT (serum glutamic pyruvic transaminase) reagent set from Pointe Scientific, Inc.

### LPS Challenge of *Sod2* ^+/−^ M*ice*

LPS was injected intra-peritoneally into 3 months-old wild type and *Sod2^+/−^ mice* at a dose of 0.01 mg/kg body weight. Plasma TNF-α levels were measured after two hours by ELISA (R&D Systems, Minneapolis, USA).

### Generation of *Mclk1/Trp53* Double Mutant Mice and Survival Study

*Trp53^+/−^* males were crossed to *Trp53^+/−^Mclk1^+/−^* females (on a C57BL/6J background), were crossed to generate the double mutant mice used in the study. *Trp53^+/−^* females, whether *Mclk1^+/+^* or *Mclk1^+/−^*, were inspected daily and weighed once a month until the end of life. The mice included in the survival analysis either lived out their natural lifespan or were humanely euthanized because they had an excessive tumor burden (>10% of body weight), or they were moribund.

### Characterization of Tumors

At necropsy, all visible tumors were dissected, weighed and processed for histological analysis. Standard H&E staining was performed to identify tumor types, according to established criteria. Weights of all sarcomas, which occurred in 45% of *Mclk1* ^+/+^ *Trp53* ^+/−^ mice and 51% of *Mclk1* ^+/−^ *Trp53* ^+/−^ mice, were expressed as percentage of body weight.

### Measurement of Muscle Wasting and Atrogenes

Measurement of muscle wasting was done at 54 weeks of age in two separate groups of *Trp53^+/−^* mice, with or without a *Mclk1* null allele. At that time, the two groups of mice had comparable body weights and no marked weight loss was noted in either group. Hind limb skeletal muscles were dissected and weighted at 54 weeks of age. Relative muscle mass was calculated as total muscle weight divided by body weight. Atrogin-1 (mouse atrophy gene 1) and MuRF1 (muscle RING finger 1) are muscle breakdown biomarkers that increase with cachexia. To measure these biomarkers, total RNA was extracted from frozen muscle using TRIzol protocol (Invitrogen). 1 µg RNA was reverse transcribed into cDNA using the QuantiTect Reverse Transcription Kit from QIAGEN. Atrogin-1 and MuRF1 mRNA content were analyzed by real-time PCR using SYBR-Green PCR Master Mix (QIAGEN) on an iCycler Real-time PCR instrument (Bio-Rad), according to the manufacturers’ instructions. Primer sequences can be provided upon request. All results were normalized to β-actin mRNA level.

### Tumor Cell Grafting

LLC1 (Lewis lung carcinoma line 1) cells were purchased from ATTC. They were cultured in DMEM (Dulbecco’s Modified Eagle’s Medium) containing 10% (v/v) FCS (fetal calf serum) and 1% antibiotics (Invitrogen). For implantation of tumor cells, *Mclk1* ^+/−^ mutants and wild type siblings (10 weeks-old males) were injected subcutaneously with 1 × 10^4^ LLC1 cells per mouse in the right supra scapular region. Tumor growth was assessed by measuring two perpendicular diameters with calipers, every other day for up to 4 weeks. The following formula was used to calculate tumor volumes: (shorter diameter)^2^ × (longer diameter)/2.

### Cytotoxic T lymphocyte (CTL) Assay

Spleens were harvested from tumor bearing mice at day 15–20 after inoculation with LLC1 cells. Splenocyte suspension was prepared by gentle disruption of the organ in a Potter homogenizer and further purified by centrifugation over lympholyte-M (Cedarlane). CD8^+^ cytotoxic T cells were isolated from spleen cells using CD8a^+^ T Cell Isolation kit II (Miltenyi Biotec). Splenic lymphocytes were restimulated *in vitro* with mitomycin C–treated LLC1 cells (100 µg/mL mitomycin C for 90 minutes; Sigma) at a 20∶1 ratio for 3 days. Recombinant human IL-2 (Sigma) was added to a final concentration of 100 IU/mL. For analysis of lytic ability, restimulated lymphocytes were co-cultured with target cells (LLC1) in 96-well plates at an effector/target ratio of 10∶1. After 4 h incubation at 37°C, the cytotoxicity was assessed using the Cytotox-ONE kit (Promega, Madison, WI, USA) according to the manufacturer's protocol. All determinations were carried out in triplicate.
